# Improving the genome editing efficiency of CRISPR/Cas9 in Arabidopsis and *Medicago truncatula*

**DOI:** 10.1007/s00425-020-03415-0

**Published:** 2020-07-08

**Authors:** Tezera W. Wolabu, Jong-Jin Park, Miao Chen, Lili Cong, Yaxin Ge, Qingzhen Jiang, Smriti Debnath, Guangming Li, Jiangqi Wen, Zengyu Wang

**Affiliations:** 1 Noble Research Institute, LLC, 2510 Sam Noble Parkway, Ardmore, OK 73401 USA; 2 Present Address: Genome Editing Naturegenic Inc, 1281 Win Hentschel Boulevard, Kurz Purdue Technology Center Suite E-1251, West Lafayette, IN 47906 USA; 3 Present Address: Guang Dong Ocean University, Faculty of Agricultural Science, #1 Haida Road, Mazhang, Zhanjiang, 524088 Guangdong China; 4 Present Address: College of Grassland Science, Qingdao Agricultural University, Changcheng Road 700, Qingdao, Shandong Province, China; 5 Present Address: College of Grassland Science, Qingdao Agricultural University, Changcheng Road 700, Qingdao, Shandong Province, China

**Keywords:** CRISPR/Cas9, Dicots, Genome editing, Mutation efficiency, Promoters

## Abstract

**Main conclusion:**

**An improved CRISPR/Cas9 system with the Arabidopsis UBQ10 promoter-driven Cas9 exhibits consistently high mutation efficiency in Arabidopsis and M. truncatula.**

**Abstract:**

CRISPR/Cas9 is a powerful genome editing technology that has been applied in several crop species for trait improvement due to its simplicity, versatility, and specificity. However, the mutation efficiency of CRISPR/Cas9 in Arabidopsis and *M. truncatula* (Mt) is still challenging and inconsistent. To analyze the functionality of the CRISPR/Cas9 system in two model dicot species, four different promoter-driven Cas9 systems to target *phytoene desaturase* (*PDS*) genes were designed. *Agrobacterium*-mediated transformation was used for the delivery of constructed vectors to host plants. Phenotypic and genotypic analyses revealed that the Arabidopsis UBQ10 promoter-driven Cas9 significantly improves the mutation efficiency to 95% in Arabidopsis and 70% in *M. truncatula.* Moreover, the UBQ10-Cas9 system yielded 11% homozygous mutants in the T1 generation in Arabidopsis. Sequencing analyses of mutation events indicated that single-nucleotide insertions are the most frequent events in Arabidopsis, whereas multi-nucleotide deletions are dominant in bi-allelic and mono-allelic homozygous mutants in *M. truncatula.* Taken together, the UBQ10 promoter facilitates the best improvement in the CRISPR/Cas9 efficiency in *PDS* gene editing, followed by the EC1.2 promoter. Consistently, the improved UBQ10-Cas9 vector highly enhanced the mutation efficiency by four-fold over the commonly used 35S promoter in both dicot species.

**Electronic supplementary material:**

The online version of this article (10.1007/s00425-020-03415-0) contains supplementary material, which is available to authorized users.

## Introduction

Among the three devised sequence-specific nucleases (SSNs) genome editing tools––zinc finger nucleases (Kim et al. 1996), transcription activator-like effector nucleases (TALENs) (Christian et al. 2010), and Clustered Regularly Interspaced Short Palindromic Repeat/CRISPR-associated protein 9 (CRISPR/Cas9)––CRISPR/Cas9 constitutes the best innovative technology that offers a great breakthrough in plant genetics and functional genomics research today. CRISPR/Cas9 has several merits, including target specificity, effectiveness, ease of use, versatility, precision, inexpensiveness, and the feasibility to allow multiple options for genome manipulation (Voytas and Gao 2014; Ma et al. 2015). Normally, CRISPR/Cas9 is made up of two crucial components: the programmed Cas9 and the customized small guide RNA (sgRNA) with 20-nucleotide homology to a DNA target site (Jinek et al. 2012; Cong et al. 2013; Mali et al. 2013). Cas9 is responsible for generating the double-strand breakages (DSBs) in the targeted DNA, while sgRNA is responsible for guiding Cas9 to the target site and forming hybridization of the RNA–DNA duplex between the sgRNA and a target sequence followed by a protospacer adjacent motif (PAM) of the genome (Li et al. 2011, 2013; Jinek et al. 2012; Wang et al. 2013). The subsequent DSBs caused by Cas9 trigger an endogenous repairing process of DNA sealing by the error-prone non-homologous end-joining (NHEJ) or homology-directed repair (HDR) pathway. The progression of repairing activity leads to deletion, insertion, or substitution at the breakage sites of coding regions, which disrupts the genes or causes frameshift (altering the reading frame), known as a gene mutation or knockout (Jinek et al. 2012; Cong et al. 2013; Mali et al. 2013). As a result of NHEJ-induced mutations, insertions/deletions (indels) of a few bases are commonly observed in CRISPR/Cas9-based plant genome editing.

The CRISPR/Cas9 genome editing system has been extensively implemented from model plants to economically important agricultural crops for trait improvement (Brooks et al. 2014; Gao et al. 2015, 2018; Michno et al. 2015; Svitashev et al. 2015; Wang et al. 2015a; Liu et al. 2017; Meng et al. 2017; Zhang et al. 2017, 2018, 2020; Hashimoto et al. 2018; Pauwels et al. 2018; Castel et al. 2019). The advancement of the CRISPR/Cas9 genome editing system through vector optimization provides opportunities for the technology to be applied toward generating desirable traits to maximize the biomass yield and/or quality for the betterment of mankind and sustainable agriculture through tackling the prevailing production constraints (Xing et al. 2014; Gao et al. 2015; Ma et al. 2015; Svitashev et al. 2015; Wang et al. 2015a; Zhang et al. 2017). Despite the effective and successful application of the CRISPR/Cas9 technology in general, there are still persisting challenges related to the low mutation efficiency in Arabidopsis and *M*. *truncatula* as compared to the improvement attained in monocot species, like rice and maize (Gao et al. 2015; Wang et al. 2015b; Yan et al. 2015; Eid et al. 2016; Mao et al. 2016; Zhang et al. 2017, 2018; Le Blanc et al. 2017; Feng et al. 2018; Pauwels et al. 2018; Castel et al. 2019). In addition, the non-reproducibility and/or inconsistency of mutation efficiency are also prevailing limitations in Arabidopsis and *Medicago truncatula* (Wang et al. 2015b; Eid et al. 2016; Mao et al. 2016; Meng et al. 2017; Curtin et al. 2018; Feng et al. 2018). The mutation efficiency of the CRISPR/Cas9 system is affected by multiple factors, including the expression level of Cas9-gRNA, the gRNA sequence, promoters driving Cas9 and small guide RNA (sgRNA), terminators, the composition of target sequence (spacer), T-DNA architecture, chromatin state, and the time frame of culture incubation (Mikami et al. 2016; Gao et al. 2018; Castel et al. 2019). In genome editing systems of dicot plants, the CaMV 35S promoter (35S) is extensively used in driving the Cas9 expression. However, studies suggested the drawback of the 35S promoter due to its high activity in somatic cells and low activity in early embryo cell division, which causes high somatic cell mutation and delayed recovery of homozygous mutation up to T2–T3 generations in Arabidopsis (Ma et al. 2015; Yan et al. 2015). Therefore, the timely high expression of Cas9 has been targeted as a key improvement for the CRISPR/Cas9 system efficiency. By replacing the 35S promoter with various tissue- or cell-specific promoters to drive Cas9, the mutation efficiency was enhanced and homozygous mutants were generated at early generations in Arabidopsis (Hyun et al. 2014; Fauser et al. 2014; Gao et al. 2015; Wang et al. 2015a; Yan et al. 2015; Mao et al. 2016; Osakabe et al. 2016; Meng et al. 2017; Tsutsui and Higashiyama 2017; Pauwels et al. 2018; Wolter et al. 2018; Zhang et al. 2018; Castel et al. 2019; Wang and Chen 2019). However, the efforts to improve the CRISPR/Cas9 efficiency so far lack consistency (non-reproducibility) and further endeavors are required to reduce somatic mutations and to increase true homozygous mutations in early generations in Arabidopsis as well as *M. truncatula* for effective edition of genes for yield and quality improvements in important legume crops.

*M. truncatula* is known as a key model legume with a small genome, diploidy, autogamy, and a short life cycle. Studies have been extensively carried out in *M. truncatula* from fundamental molecular, physiological, and developmental mechanisms to translate and applied trait improvement in related economically important legume crops (Michno et al. 2015; Čermák et al. 2017; Curtin et al. 2017, 2018; Meng et al. 2017; Gao et al. 2018). To further promote the advancement of functional genomics studies in line with the emerging cutting-edge genome editing technology, a CRISPR/Cas9 system with high precision and efficacy in such an important model legume is highly desired. A few CRISPR/Cas9 genome editing activities have been performed in *M. truncatula*. For instance, the optimized soybean codon-Cas9 driven by the 35S promoter in *M. truncatula* using hairy root transformation by targeting GUS transgenic lines successfully abolished the GUS expression (Michno et al. 2015). Likewise, by targeting *MtPDS*, 10% mutation efficiency was reported using the 35S promoter-driven Cas9 in *M. truncatula* (Meng et al. 2017). Additionally, using the soybean GmUbi promoter to drive Cas9, a 50–70% mutation efficiency was reported by targeting multiple genes (*PHO2*-like and *PEN3*-like) in *M*. *truncatula* (Curtin et al. 2018). However, using the same construct (GmUbi promoter-driven Cas9) to target a small RNA processing gene (*MtHen1*) generated mutants with reduced mutation efficiencies as well as low mutation recovery (Curtin et al. 2018). During the preparation of this manuscript, Zhang et al. (2020) reported the efficient generation of homozygous/bi-allelic mutants in *M. truncatula* using a hairy root system in the second generation but with low mutation efficiency (recovery rate) in the first generation. Therefore, the mutation efficiency of the CRISPR/Cas9 system so far achieved in Arabidopsis and *M. truncatula* still shows remarkable inconsistency, suggesting that more efforts in advancing genome editing tools with high precision and efficacy in these species are necessary.

In search for optimal promoters to drive the expression of Cas9 as an important factor for high-efficiency genome editing, four CRISPR/Cas9 modules for mutation efficiency improvement by targeting the Cas9 expression using different promoters were constructed. Four promoter-driven Cas9 cassettes––namely, UBQ10-Cas9, EC1.2-Cas9, AMGE3-Cas9, and 35S-Cas9––were tested in *Arabidopsis thaliana* and *M. truncatula* by targeting *phytoene desaturase* genes (*AtPDS3* and *MtPDS)*. Typically, mutants of the *PDS* gene exhibit a visible phenotype (albino and/or mosaic) that allows screening and characterization of null mutants at seedling or callus stages. Multiple mutants for both species were generated and subjected to phenotypic and genotypic analyses. The overall results revealed that the UBQ10 promoter-driven Cas9 significantly improves the mutation efficiency in Arabidopsis (95%) and *M. truncatula* (70%) at T1 and T0 generations, respectively. Moreover, the UBQ10-Cas9 vector yielded 11% homozygous mutants in the T1 generation in Arabidopsis. Consistently, the UBQ10-Cas9 vector highly enhanced the mutation efficiency by four-fold over the commonly used 35S promoter in both species.

## Materials and methods

### Plant growth conditions

*Arabidopsis thaliana* ecotype Columbia (Col-0; seeds from ABRC, Ohio State University) and *Medicago truncatula* (R108; Noble Research Institute) were used for transformation. All generated transgenic lines and wild-type plants were grown under growth chamber or greenhouse conditions with 22/19 °C day/night temperature, 16/8 h day/night photoperiod, 150 µmol m^−2^ s^−1^ light intensity, and 70–80% relative humidity.

### Construction of new CRISPR/Cas9 vectors and generation of transgenic plants

**Fig. 1:**
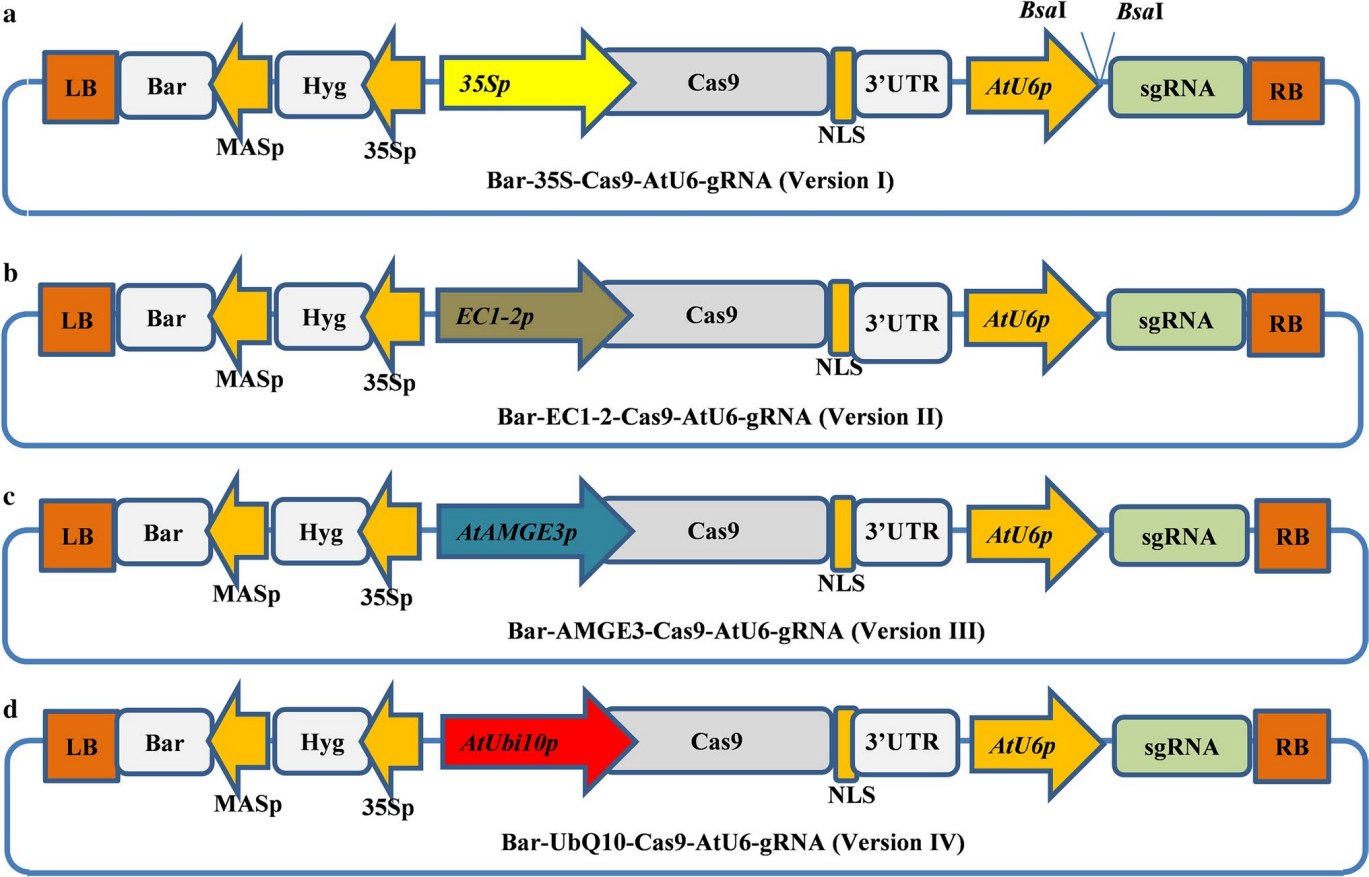
Illustration of schematic diagrams of new CRISPR/Cas9 vectors based on the pCAMBIA backbone for *AtPDS3* and *MtPDS* gene editing in Arabidopsis and *M. truncatula*. **a** CRISPR vector with Cas9 driven by the 35S promoter (version I). **b** CRISPR vector with Cas9 driven by the EC1-2 promoter (version II). **c** CRISPR vector with Cas9 driven by the AMGE3 promoter (version III). **d** CRISPR vector with Cas9 driven by the UBQ10 promoter (version IV)

The pCAMBIA backbone was used to construct new CRISPR/Cas9 vector modules with specific promoters, such as Bar-Hyg-35S-Cas9-AtU6-gRNA, Bar-Hyg-EC1.2-Cas9-AtU6-gRNA, Bar-Hyg-AMGE3-Cas*9*-AtU6-gRNA, and Bar-Hyg-UBQ10-Cas9-AtU6-gRNA, with such short names as vector versions I, II, III, and IV, respectively (Fig. 1a–d). The vector backbone was digested by *Hind* III and *Eco*R I and ligated with MAS promoter-*Bar*-MAS terminator-*Nco* I site. As a result, the *Bar*-vector was digested by *Nco* I, and then the linear vector was ligated with the *SpyCas9* gene. The ligated product, Bar-SpyCas9 construct, was added by the AtU6-small guide RNA (sgRNA) complex at the *Eco*R I site. The enzyme site, *Nco* I, between the Bar and SpyCa9 genes was used for cloning all promoters: 35S, EC1.2, AMGE3, and UBQ10. The promoters of the three Arabidopsis genes (EC1.2, AMGE3 and UBQ10) were amplified by PCR from the Arabidopsis genomic DNA with specific primers (Table S1). Then each promoter was assembled into the CRISPR/Cas9 backbone using Golden Gate cloning, restriction enzyme digestion and the PCR parallel overlapping extension system in a stepwise approach. The subsequent new binary vectors with *AtPDS3- or MtPDS-*gRNA-CRISPR/Cas9 modules were transformed into *Agrobacterium tumefaciens* strains GV3101 for Arabidopsis and EHA105 for *M. truncatula.* Plant transformation was achieved by *Agrobacterium*-mediated transformation delivery systems. Briefly, for *M. truncatula* transformation, fully developed trifoliate leaves were collected from 4-week-old plants and sterilized. Trifoliate leaf disc explants were infected with respective *Agrobacterium tumefaciens* strain, incubated for 24–36 h in the dark at 24 °C and then transferred onto selection media containing phosphinothricin for callus induction. Resistant calli were produced and proliferated in 5–6 weeks. The resistant calli were then transferred onto a regeneration medium and cultured under light conditions of 150 µmol m^−2^ s^−1^ at 24 °C with a 16/8 h photoperiod. PCR verification of the regenerated transgenic plants with good roots was conducted with genomic DNA extracted from leaf tissues using BAR gene primers. Arabidopsis was transformed using the floral dipping method (Clough and Bent 1998).

### Phenotypic screening of *atpds3* and *mtpds* mutants generated by different CRISPR/Cas9 systems

*atpds3* and *mtpds* mutants were easily identified by their albino or mosaic phenotypes at the seedling (*atpds3* and *mtpds*) or regeneration stage (*mtpds*). Furthermore, the Arabidopsis mosaic mutants were classified based on the proportion of albino to greenness in the leaves. Thus, the phenotypic analysis of each vector was determined based on the proportion of albino/mosaic phenotype to normal (wild-type) phenotype. This analysis provides preliminary mutation efficiency for different vector modules.

### Genotyping mutants generated by CRISPR/Cas9 systems in Arabidopsis and *M. truncatula*

To screen putative CRISPR/Cas9-generated mutants of *AtPDS3* and *MtPDS*, genomic DNA (gDNA) was extracted from young leaves of transgenic lines using a CTAB method (Rogers and Bendich 1994). Then the target region of the gene was amplified by PCR with specific primers designed from the borders of the target site (Table S1). The PCR products were treated by 1 µl of Antarctic Phosphatase and 0.25 µl Exonuclease I and incubated in a thermal cycler at 37 °C for 1 h, 80 °C for 20–30 min, and 10 °C hold to clean up PCR products by eliminating dNTP residues. Then 5 µl of PCR products was used for Sanger sequencing analysis to verify CRISPR/Cas9-induced mutations in transgenic lines. The transgenic lines with double peaks of chromatographs were considered as putative candidate mutants with respect to the targeted genes of each species. The ratio of mutagenized lines versus non-mutant lines across the examined populations was used as the mutation efficiency. Furthermore, to identify and characterize the nature of mutation types induced by different CRISPR/Cas9 systems at the target sites, the amplified fragments of mutated regions were sub-cloned into the pGEM-T Easy vector (Promega, Madison, WI, USA). To precisely identify the mutation events (indels and/or substitution) at the target site, 15–20 colonies were randomly selected for plasmid DNA isolation. The isolated single-colony plasmids were subjected to Sanger sequencing for mutation-type determination as well as for homozygous, heterozygous, and/or chimeric mutant assessment. Reads were analyzed by aligning with the reference sequence using the SeqMan Pro 16.0 (DNASTAR software, https://www.dnastar.com/quote-request/).

## Results and discussion

CRISPR/Cas9 is a cutting-edge programmable genome editing technology using sequence-specific nucleases (SSNs) through site-directed recognition and precise cleavage of DNA. Several previous studies recommended the importance of CRISPR/Cas9 optimization using the appropriate promoter to drive the Cas9 expression as an important factor to achieve high efficiency in Arabidopsis genome editing (Hyun et al. 2014; Wang et al. 2015a; Yan et al. 2015; Eid et al. 2016; Mao et al. 2016). Therefore, improving stable mutagenesis efficiency of CRISPR/Cas9 vectors is still necessary in dicots in general, and in Arabidopsis and *M. truncatula* in particular (Brooks et al. 2014; Liu et al. 2017; Zhang et al. 2017, 2018, 2020; Pauwels et al. 2018; Castel et al. 2019).

**Fig. 2:**
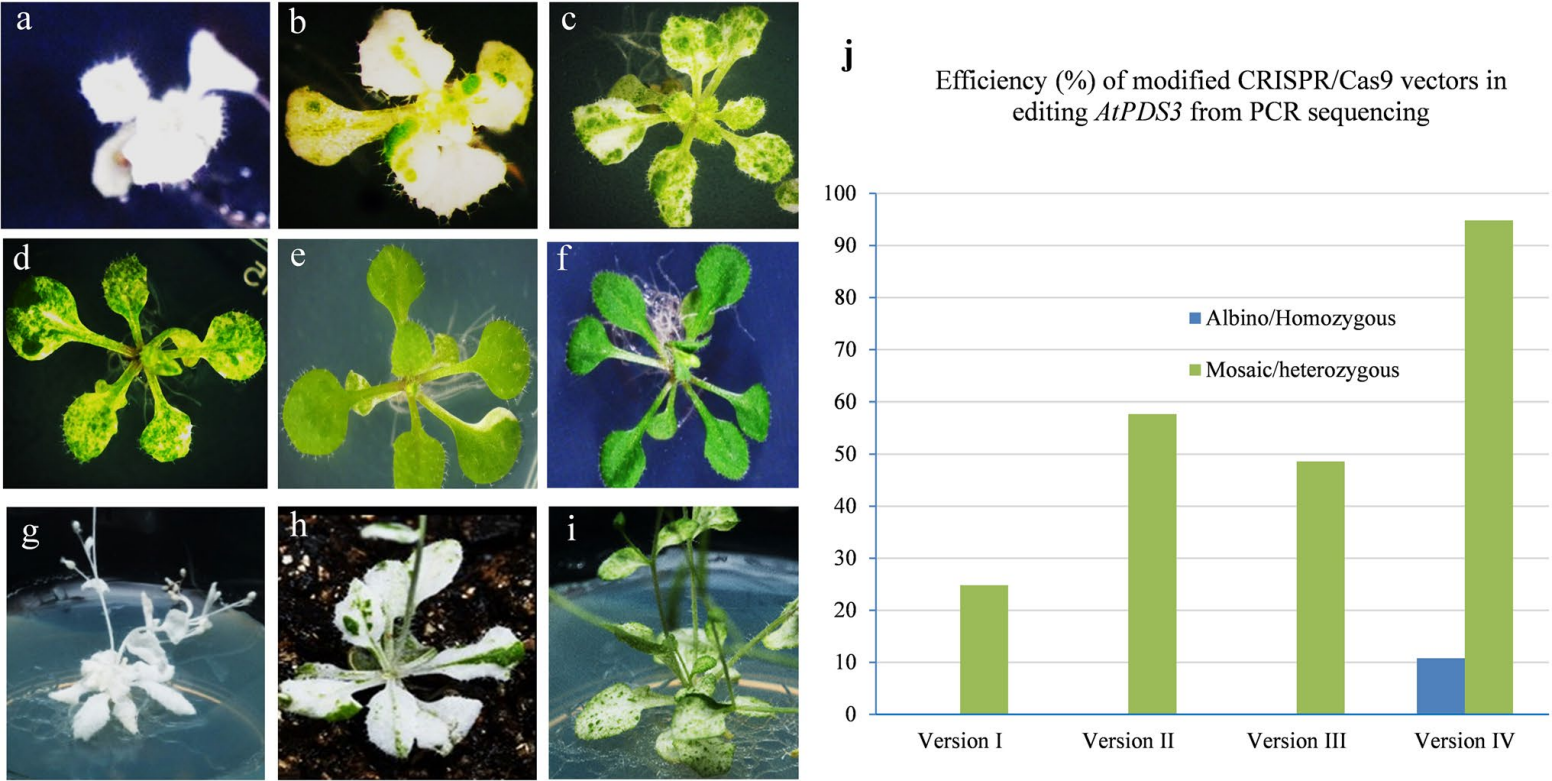
Phenotypes of Arabidopsis *atpds3* mutants generated by different CRISPR/Cas9 vectors in T1 generation. **a** Phenotype of albino/homozygous *atpds3* mutant generated by vector version IV at the seedling stage. **b** Phenotype of mosaic/chimeric *atpds3* mutant generated by vector version IV at the seedling stage with albino (white) dominant over greenness. **c** Phenotype of mosaic/chimeric *atpds3* mutant generated by vector version IV at the seedling stage with almost equal albino (white) versus greenness distribution on leaf blade. **d, e** Phenotype of mosaic/chimeric *atpds3* mutant representing greenness dominant over albino, which was observed across all tested vector versions. **f** Phenotypes representing heterozygous or normal wild type at the seedling stage. **g–i** Phenotypes of *atpds3* mutants at flowering stage. **j** Graph depicting the overall mutation efficiency of *atpds3* mutants generated from CRISPR/Cas9 vector versions I, II, III, and IV in Arabidopsis. Note: Complete albino (homozygous) and the intensity of albino over greenness were only detected in new vector version IV

**Fig. 3:**
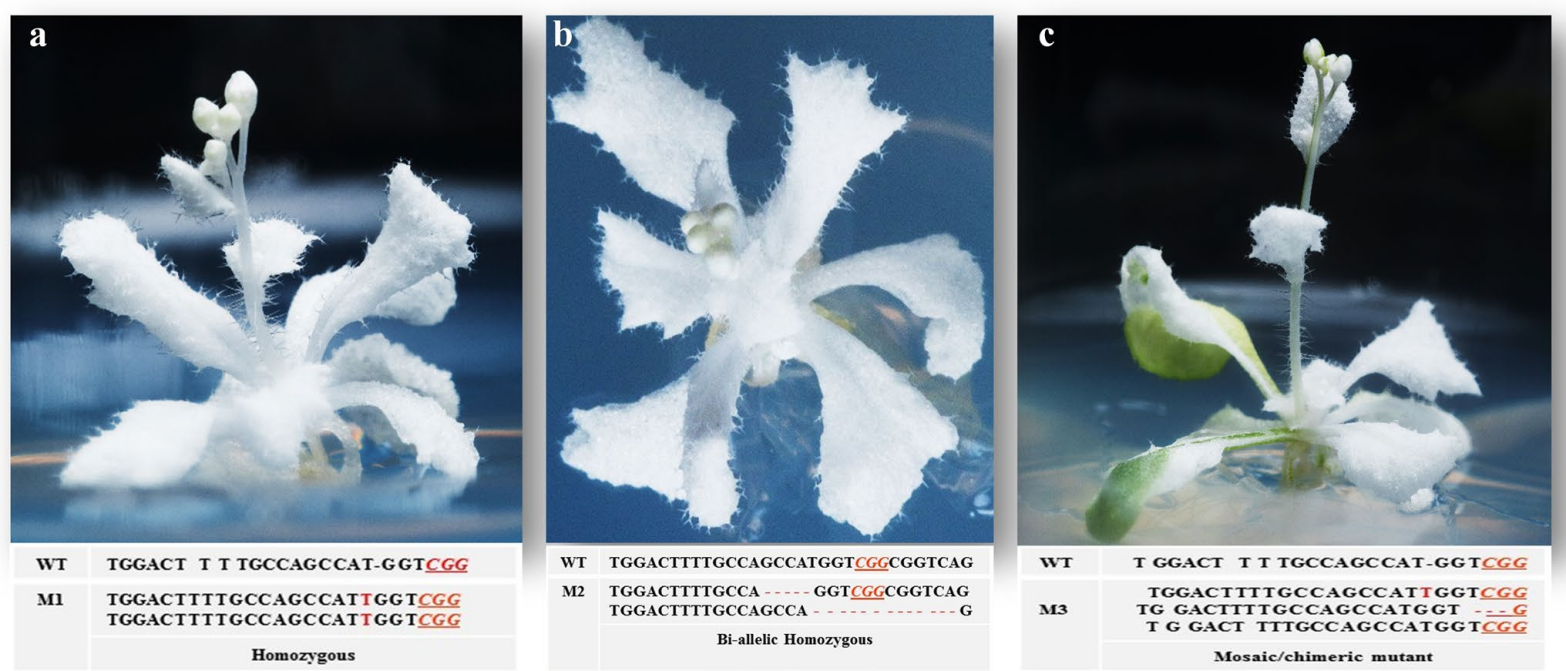
Phenotype of *atpds3* mutants generated by the CRISPR/Cas9 vector version IV at T1 generation. **a** Phenotype of albino *atpds3* mutant with mono-allelic homozygous mutation of single base T insertion at the flowering stage. **b** Phenotype of albino *atpds3* mutant with bi-allelic homozygous mutation of different-nucleotide deletion at the flowering stage. **c** Phenotype of *atpds3* mosaic/chimeric mutant at flowering stage with single base insertion, three bases deletion, and wild-type sequence events. Note: the PAM sequence is indicated by red underlined font. Deletions are indicated by “-” and insertions are shown in red font. WT, wild type; M1, M2, and M3 indicate mutant lines 1, 2, and 3

**Fig. 4:**
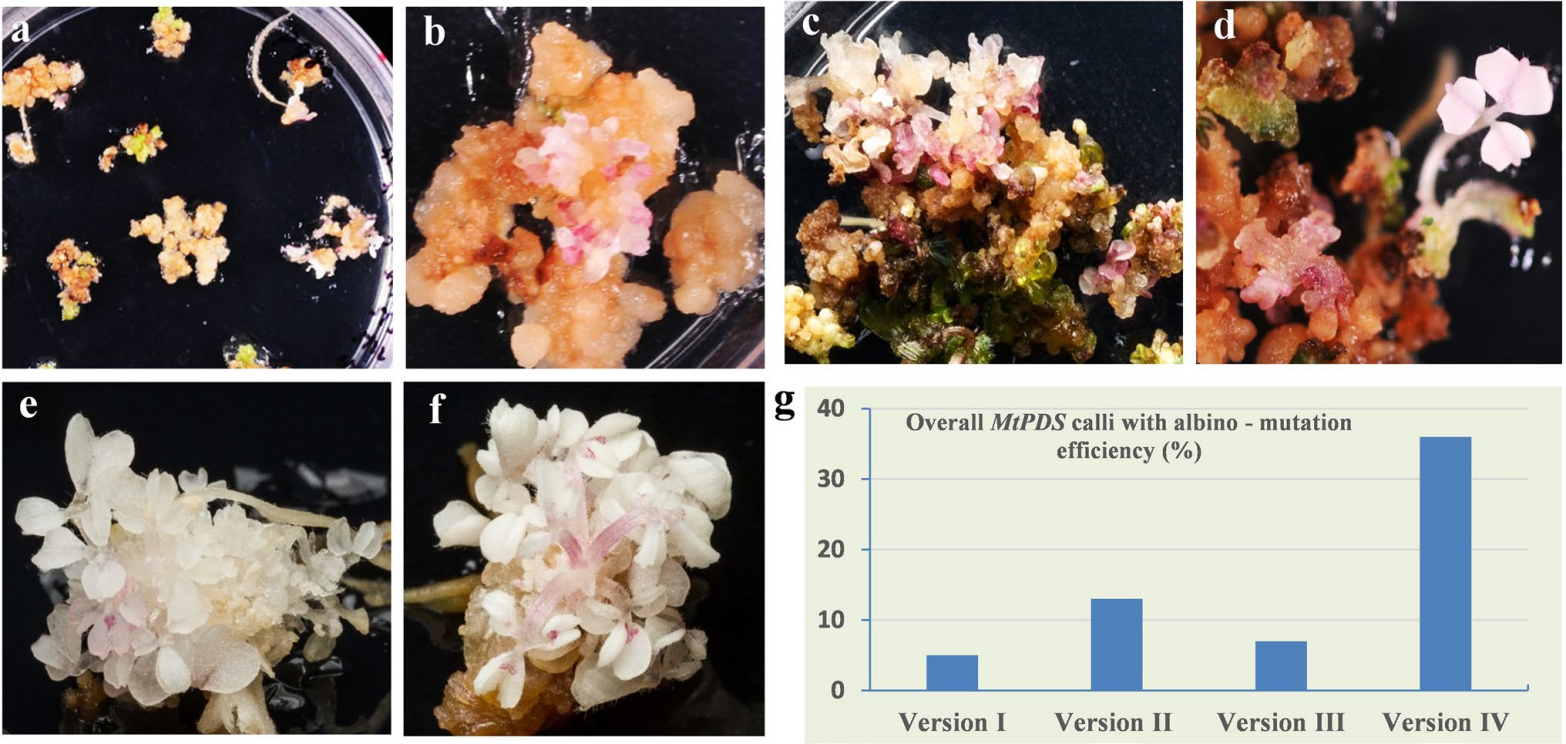
Phenotype of *mtpds* mutated calli at different stages generated by new CRISPR/Cas9 vector versions at T0 generation. **a, b** First stage of mutated calli of *mtpds* on culture medium. **c** Second stage of mutated calli of *mtpds* on culture medium. **d–f** Mutated calli of *mtpds* at the regeneration stage on culture medium. **g** Graph of mutated calli at regeneration (third stage) stage showing the mutation efficiency of four new CRISPR/Cas9 vector versions I, II, III and IV

In this study, the CRISPR/Cas9 vector cassettes (versions) were systematically modified with four different promoters (35S, EC1.2, AMGE3, and UBQ10) to drive the Cas9 expression and tested the genome editing efficiency in Arabidopsis and *M. truncatula* by targeting *AtPDS3* and *MtPDS.* For convenience, these constructs were named versions I, II, III, and IV, respectively (Fig. 1a–d). The previously reported candidate gRNAs (spacers) for Arabidopsis (Li et al. 2013) and *M. truncatula* (Meng et al. 2017) were used, since their effectiveness and specificities were proven. The vectors were introduced into Arabidopsis and *M. truncatula* via *Agrobacterium*-mediated transformation. To determine the mutation efficiency in Arabidopsis *AtPDS3* gene editing, 582 transgenic lines were generated from different CRISPR/Cas9 vectors. From noticeable phenotypes, such as albino, mosaic, and wild type, the mutation efficiency of each vector was determined. The results revealed significant differences among tested vectors, with 11% (17 out of 157), 26% (43 out of 163), 34% (36 out of 105), and 69% (109 out of 157) plants showing phenotypes for vector versions I, II, III, and IV, respectively (Table 1). The highest phenotype-based mutation efficiency was observed in vector version IV, at 69%. Interestingly, vector version IV also yielded 11% mutants with a complete albino phenotype in the T1 generation (Fig. 2a, Fig. 3a–c, Table 1). Furthermore, based on the albino/mosaic phenotypes, the mutants were qualitatively categorized into five groups: (1) mutants with complete albino phenotype (Fig. 2a, g); (2) mutants with albino dominant over greenness phenotype (Fig. 2b, h); (3) mutants with albino almost equal to greenness phenotype (Fig. 2c, i); (4) mutants with greenness dominant over albino phenotype (Fig. 2d, e); and (5) wild-type-like phenotype (normal) (Figs. 2f, S1, Table 1). The albino/mosaic mutants generated by version IV was dominant over greenness compared to other vector versions (Table 1). It was worth noting that the mutation efficiency was assessed based on the phenotypes in the T1 generation. However, for the promoters EC1.2 and AMGE3, it is expected that only in the T2 generation, the effect of the expression in egg cells or during meiosis would be visible and resulting in large number of partly or completely mutated progeny, though this was not tested. These phenotypic categories were consistently observed not only at the vegetative stage but also at the reproductive stage with albino/mosaic stems, flower buds, and siliques (Figs. 2g–i, S2a–d). Furthermore, putative *atpds3* mutants were genotyped to assess the overall mutation efficiency of the modified vector modules at the molecular level. Here, two genotyping approaches were employed: (1) amplifying the target region by PCR and analyzing by Sanger sequencing and (2) cloning the amplified PCR products into pGEM-T Easy vector (TA-cloning) followed by Sanger sequencing. The transgenic lines (albino, mosaic, and wild type) generated from each vector were subjected to the first step of mutation efficiency determination by amplifying the target region (326 bp in size) followed by direct sequencing of the PCR amplicons. The results showed a mutation efficiencies of 25%, 58%, 50%, and 95% for vector versions I, II, III, and IV, respectively (Fig. 2j). As expected, vector version IV showed the highest overall mutation efficiency (95%), which was nearly four-fold of the commonly used vector version I (Fig. 2j). To further characterize the nature of mutations occurred in mutants with albino, mosaic, or normal phenotypes, TA cloning followed by sequencing analysis was performed using mutant lines from vector version IV. Fifteen individual mutants with complete albino, mosaic, or wild-type phenotypes were used for this analysis. Deletions, insertions, and substitutions were detected, among which single-nucleotide insertion is the most frequent mutation (34%) across tested mutants (Fig. 3a, c, Table 2). Nucleotide T insertion occurred at a 20% frequency, followed by nucleotide A insertion at 10% occurrence frequency (Table 2). Nucleotide deletions (2–28 bp) were detected with lower occurrence frequency. Most mutation events occurred at three nucleotides upstream of the PAM (73%), except that the 28 bp deletion occurred in both sides of the PAM (Table 2). These results were consistently in agreement with previous studies (Pan et al. 2016; Tian et al. 2017; Klimek-Chodacka et al. 2018; Pauwels et al. 2018; Zhu et al. 2018). In general, both phenotypic and genotypic analyses clearly showed that the mutation efficiency of vector version IV prevails the other vectors. Recently, a CRISPR/Cas9 system for efficient generation of free multiplex mutants in Arabidopsis was reported (Wang and Chen 2019). The authors compared the efficiency of Cas9 driven by UBQ10 and 35S promoters and found that UBQ10 is by far better than the 35S promoter with homozygous mutants at the T1 generation (Wang and Chen 2019).

**Table 1:** Arabidopsis *atpds3* mutants from different promoter-driven Cas9 are classified into five phenotypic categories based on albino (whitish) versus greenness of leaf blade at the seedling stage at T1 generation

Mutant label	Mutant phenotypic category	No of examined transgenic lines in four vector versions			
		I	II	III	IV
1 (A)	Albino	0	0	0	17
2 (B)	Mosaic (White > Green)	0	0	0	26
3 (C)	Mosaic (White = Green)	2	16	13	24
4 (D and E)	Mosaic (Green > White)	15	27	23	42
5 (F)	Normal (WT/heterozygous)	140	120	69	50
	Total Transgenic lines of T1 generation	157	163	105	159

**Table 2:**
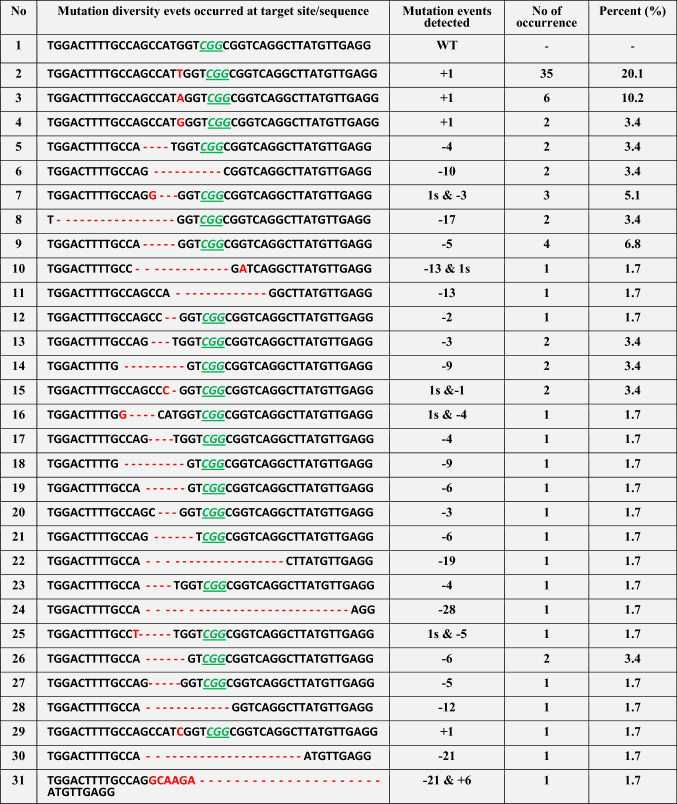
Diverse mutation events occurred at the target site of *AtPDS3* gene (gRNA1) in 15 independent mutants generated using the CRISPR/Cas9 vector version IV

Wild-type (WT) sequence of target site; deletions are indicated by red dashed lines, insertions or substitutions are indicated by red font with plus ‘ + ’ and “s”s sign, respectively; PAM (CGG) is indicated by green underlined italicized font. Mutation events were analyzed using TA-cloning and Sanger sequencing

**Fig. 5:**
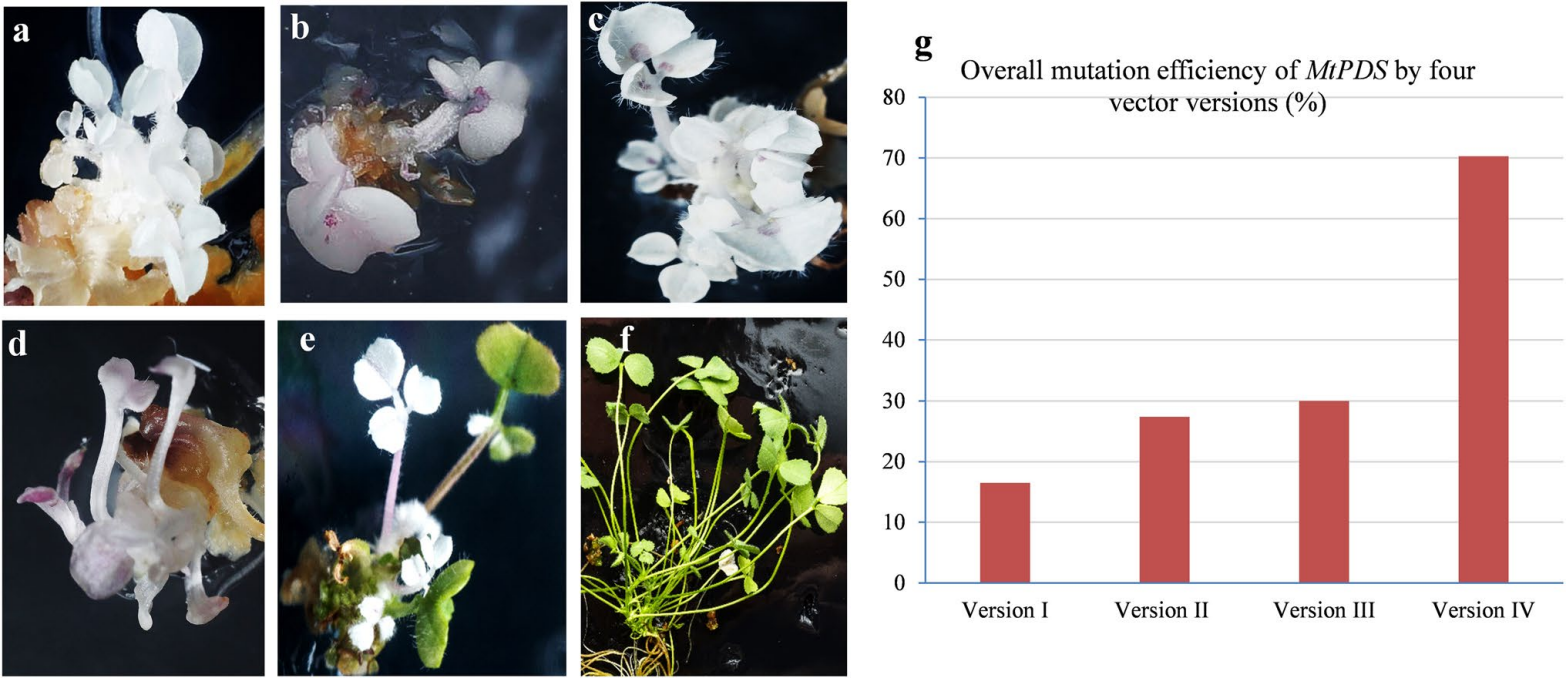
Phenotype of *mtpds* mutants generated by new CRISPR/Cas9 vectors driven by different promoters in T0 generation. **a** Phenotype of *mtpds* albino mutant generated by vector version IV. **b** Phenotype of *mtpds* albino mutant generated by vector version I. **c** Phenotype of *mtpds* albino mutant generated by vector version III. **d** Phenotype of *mtpds* albino mutant generated by vector version III. **e** Phenotype of *mtpds* mosaic mutant generated by vector version IV. **f** Phenotype of wild type. **g** Graph of overall efficiency of *mtpds* mutants generated by new CRISPR/Cas9 vector versions I, II, III, and IV

**Fig. 6:**
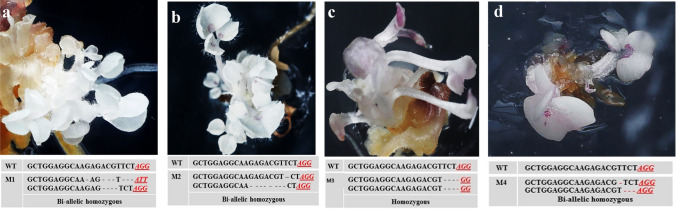
Phenotypic and genotypic analyses of *mtpds* mutants generated by new CRISPR/Cas9 vector versions (I–IV). **a** Phenotype of *mtpds* bi-allelic homozygous mutant generated by vector version IV. **b** Phenotype of *mtpds* bi-allelic homozygous mutant generated by vector version II. **c** Phenotype of *mtpds* mono-allelic homozygous mutant generated by vector version III. **d** Phenotype of *mtpds* bi-allelic homozygous mutant generated by vector version I. Note: the PAM sequence is indicated by red underlined font; mutation events deletions are indicated by “-” and substitutions are shown in red font. WT, wild type; M1, M2, M3, and M4 indicate mutant #1, #2, #3, and #4, respectively

Despite the importance of *M. truncatula* as a key model legume for basic and applied research in legumes, application of the CRISPR/Cas9 technology is less effective than in cereal model species (Michno et al. 2015; Čermák et al. 2017; Curtin et al. 2017, 2018; Meng et al. 2017; Zhang et al. 2020). New multiplex genome editing modules showed a wide range of mutation efficiency in *M. truncatula* by targeting three nodule-specific cysteine-rich (NCR) peptide genes and producing triple (6.5%), double (28%), and single mutants (59%) (Čermák et al. 2017). These studies suggested the advantage of multiplex approach over single gRNA system in inducing loss-of-function mutations (Čermák et al. 2017; Zhang et al. 2017). It has also been reported that a 50–70% CRISPR/Cas9 mutation efficiency was achieved by the soybean GmUbi promoter-driven Cas9. When *PHO2*-like and *PEN3*-like genes were targeted for editing, homozygous mutations were successfully induced in multiple independent T0 plants in *M*. *truncatula* (Curtin et al. 2017). However, similar CRISPR/Cas9 vector modules, when a small RNA processing gene (*MtHen1*) was targeted, reduced mutation efficiencies with low mutation recovery were yielded, suggesting inconsistency of the system (Curtin et al. 2018). Therefore, CRISPR/Cas9 vector optimization in this model species is still an ongoing research goal (Curtin et al. 2017, 2018; Pauwels et al. 2018; Wang and Chen 2019; Castel et al. 2019; Zhang et al. 2020). This process is in line with these objectives to enhance the genome editing opportunity in dicots. To assess the functionality and efficiency of each vector in *M. truncatula*, the same set of CRISPR/Cas9 vector modules was used (versions I, II, III, and IV) to target *MtPDS*. Since *mtpds* mutants were generated through callus induction and regeneration, the mutation efficiency was evaluated at two stages: The first evaluation of mutation efficiency was carried out at callus subculture and regeneration stages, as mutated callus was easily identifiable by the albino/mosaic phenotypes. Three assessments were made throughout the stages of callus subculture and regeneration with 3-week intervals (Fig. 4a–f). The mutation efficiency showed an increasing trend from vector version I to version IV and from the first stage to the third stage of callus subculture and regeneration (Table 3). At the third stage, the overall evaluation of mutated calli showed a mutation efficiency of 5%, 13%, 7%, and 36% for vector versions I, II, III, and IV, respectively (Fig. 4g, Table 3). The albino phenotype in calli from the improved vector version IV showed the highest percentage, with 18%, 30%, and 36% at the first, second, and third stages of callus subculture, respectively, indicating high effectiveness of this vector as compared to other vectors (Fig. 4g, Table 3). At the subsequent plantlet development stages (Fig. 4d–f), a similar mutation efficiency evaluation process for each vector was followed. Similar as in Arabidopsis, three categories of mutants were also defined as albino, mosaic, and wild-type lines (Figs. 5a–f, S5a–f) in *M. truncatula.* However, three mosaic/chimeric mutants were only observed across the regenerated lines in two vector versions (Figs. 5e, S3e–f), in contrast to high somatic mutation in Arabidopsis. To further determine the overall mutation efficiency of each vector, DNA from the whole mosaic leaves was isolated and the 389 bp fragment spanning the target region of *MtPDS* by PCR for Sanger sequencing was amplified. In addition to the lines with albino/mosaic phenotypes, a total of 15, 50, 13, and 41 independent lines with wild-type-like phenotypes by sequencing analysis for vector versions I, II, III and IV, respectively, were genotyped. The overall mutation efficiencies were 16.5%, 27%, 30%, and 70% for vector versions I, II, III, and IV, respectively (Fig. 5g). Remarkably, vector version IV showed the highest overall mutation efficiency, which was four-fold of vector version I. Similar as in Arabidopsis, vector version IV consistently showed the highest mutation efficiency at both callus and plantlet stages (Fig. 5g). To further characterize the nature of mutation events in regenerated lines with albino, mosaic, or wild-type phenotypes, TA cloning followed by sequencing analysis was also performed in 15 representative mutants (Table 4). As expected, various types of mutations were detected in these mutants with homozygous, heterozygous, or chimeric mutations across tested vector versions (Table 4). The sequencing analysis also showed mono-allelic and bi-allelic homozygous mutants with varied nucleotide deletions (1–11 bp) (Fig. 6a–d, Table 4).

**Table 3:** Number of mutated albino calli of *mtpds* examined at different callus developmental stages using four vector versions

Promoter	Number of calli examined	*MtPDS* mutated albino calli efficiency (%) at three calli developmental stages		
		1st stage	2nd stage	3rd stage
Version I	207	2	3	5
Version II	274	3	10	13
Version III	179	4	6	7
Version IV	230	18	30	36

**Table 4:**
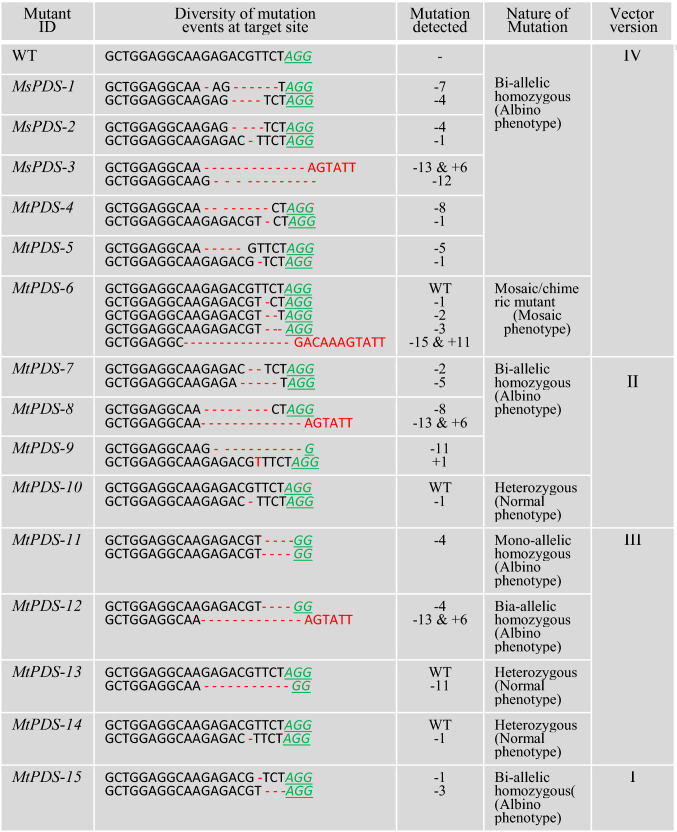
Diverse mutation events occurred at the target site of *MtPDS* gene (gRNA1)

Wild-type (WT) sequence of target site; deletions are indicated by red dashed lines; insertions or substitutions are indicated by red font with plus “ + " and “s” sign, respectively; PAM is indicated by green underlined italicized font. Mutation events in each mutant line were confirmed using TA-cloning and Sanger sequencing analysis

The inheritance of mutation events detected in T1 (Arabidopsis) or T0 (*M. truncatula*) generation in order to confirm the transmission of the mutations to the next generation by self-pollination in both species was also assessed. The results showed that mosaic mutants in both species harbor heterozygous mutations and segregate seedlings with complete albino in the next generation, which have identical mutation nature as their parents. It was further explored whether different mosaic phenotypes in Arabidopsis *atpds3* mutants, as shown in Fig. 2a–d, are inheritable in subsequent generations. Thus, mutants generated by vector version IV with different levels of mosaic phenotypes were selected: (1) mutant phenotype with albino dominant over greenness, (2) mutant phenotype with albino equal or close to greenness, and (3) mutant phenotype with albino less than greenness. The individual plants were self-pollinated, and seeds were harvested to confirm the inheritability of mutations in the next generation. 300 seeds were randomly selected from each line and planted on half-strength MS medium for segregation analysis. The proportion of complete albino mutants in categories (1) and (2) was significantly higher than that in category (3) (Fig. S4a–h). The proportion of complete albino (homozygous) progenies was directly correlated with the mosaic intensity in the parental lines (Fig. S4a–f). In contrast, the mosaic mutant with greenness dominant phenotype segregated more wild-type-like plants in the next generation (Fig. S4g, h). In a similar manner, the inheritability of regenerated *M. truncatula* heterozygous *MtPDS* mutants from vector version IV was also examined (Fig. S5a–f). First, the heterozygous mutants were identified by sequencing analysis from the T0 generation with one allelic mutation event (Fig. S5a, c). Then, the selfed T1 seeds were grown on half-strength MS medium. Obviously, both heterozygous mutants produced seedlings with complete albino segregating progenies (Fig. S5b, d). Furthermore, TA cloning followed by sequencing analysis was performed to determine whether mutation events in the parent line are detected in the progenies. Matching mutation events were detected in tested progenies as identical to the parental lines (Fig. S5e, f).

Taken together, the improved CRISPR/Cas9 vector version IV (UBQ10-Cas9) consistently showed effectiveness in improving the CRISPR/Cas9 mutation efficiency in both Arabidopsis and *M. truncatula* using a single gRNA approach. It has been reported that the UBQ10 promoter facilitates moderate expression in nearly all tissues (Norris et al. 1993) and provides stability to enable reliable transformation in Arabidopsis (Grefen et al. 2010). It has been also suggested that the high mutation efficiency achieved by the UBQ10 promoter is due to the high expression of Cas9 at early embryonic development (Wang and Chen 2019), which is in agreement with these results. This result is also consistently in agreement with recent mutation frequency improvement made in Arabidopsis using UBI4-2/UBI10 promoter-driven Cas9 (Fauser et al. 2014; Pauwels et al. 2018; Wolter et al. 2018; Castel et al. 2019; Wang and Chen 2019; Zhang et al. 2020). In monocot genome editing, like maize and rice, cereal ubiquitin promoters are the most preferred promoters in driving Cas9 expression due to their superior performance compared to the 35S promoter (Xie et al. 2015; Wang et al. 2016). By using a multiplex gRNA approach, the improved vector UBQ10-Cas9 also enhances the mutation efficiency in tetraploid alfalfa, with 44–75% mutation efficiency in editing several genes (Wolabu et al., unpublished data). In conclusion, this improved CRISPR/Cas9 system consistently demonstrates the enhancement of mutation efficiency of genome editing in both Arabidopsis and *M. truncatula*. This improved system might be applicable in other legumes, like alfalfa.

### *Author contributions statement*

TW, ZW, and JW conceived the experiments and wrote the manuscript; TW, JJP, MC, and LC are involved in optimizing the vectors, experimental execution, and data analysis; YG, QJ, GL, and SD contributed to plant transformation.

## Electronic supplementary material

Below is the link to the electronic supplementary material.

Supplementary file1 (DOCX 836 kb)

## Abbreviations

AMGE3: Meiosis-specific promoter
AtPDS3: *Arabidopsis thaliana* PDS
EC1.2: Egg cell-specific
PAM: Protospacer adjacent motif
PDS: Phytoene desaturase
UBQ10: Ubiquitin10
