# Supplementary material for: Improving the genome editing efficiency of CRISPR/Cas9 in Arabidopsis and Medicago truncatula
Source: Planta. 2020 Jul 8;252(2):15. doi: 10.1007/s00425-020-03415-0 (PMC7343739; doi:10.1007/s00425-020-03415-0)
Supplement: Supplementary file 1 — Supplementary file1 (DOCX 836 kb) [file 425_2020_3415_MOESM1_ESM.docx]

**Supplementary Materials**

**Improving the genome-editing efficiency of CRISPR/Cas9 in Arabidopsis and *Medicago truncatula***

Tezera W. Wolabu^*^, Jong-Jin Park^1*^, Miao Chen^2^, Lili Cong^3^, Yaxin Ge, Qingzhen Jiang,

Smriti Debnath, Guangming Li, Jiangqi Wen^**^ and Zengyu Wang^3**^

Noble Research Institute, LLC, 2510 Sam Noble Parkway, Ardmore, OK 73401, USA

**Figure S1**

**
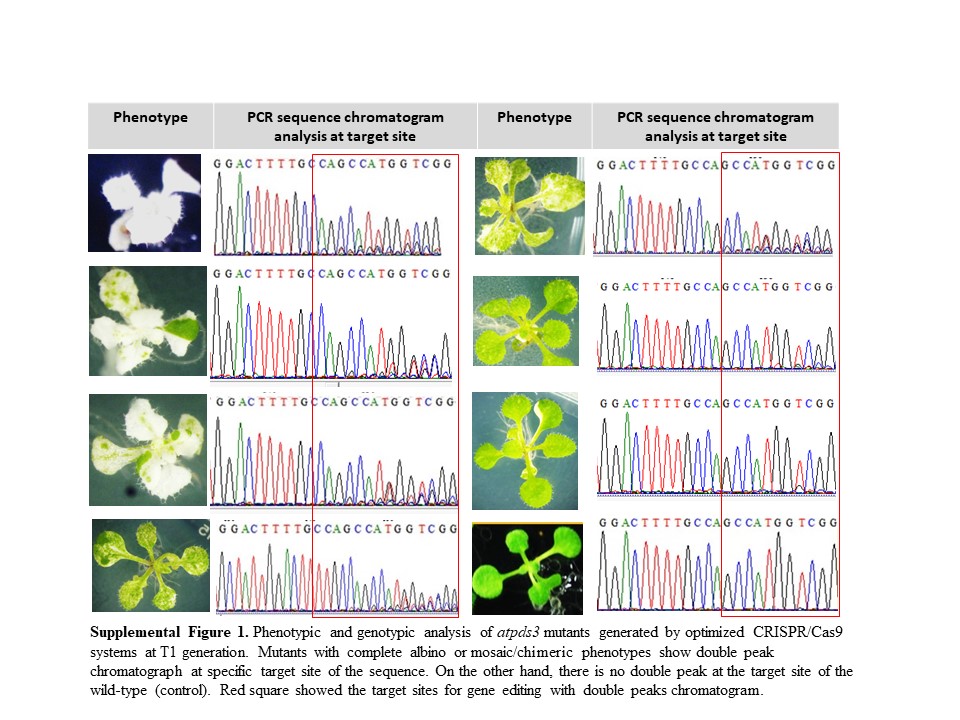
**

**Figure S1** Phenotypic and genotypic analysis of *atpds3* mutants generated using the modified CRISPR/Cas9 systems at T1 generation. Mutants with complete albino or mosaic/chimeric phenotypes show double peak chromatograph at specific target site of the sequence. On the other hand, there is no double peak at the target site of the wild-type (lower right panel). Red square showed the target sites for gene editing with double peaks chromatogram.

**Figure S2**

**
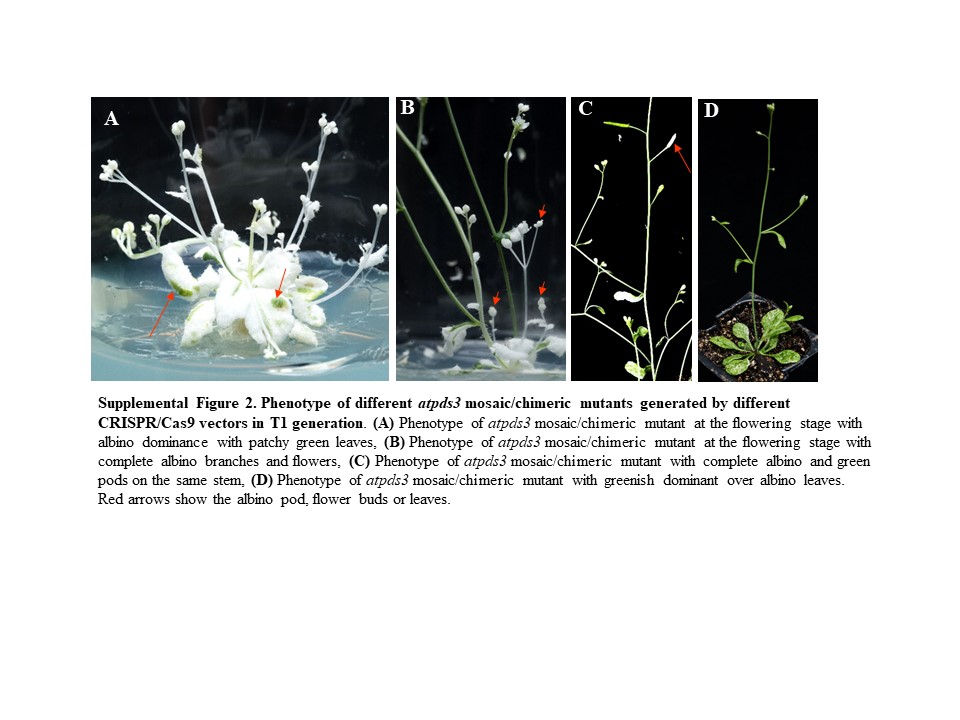
**

**Figure S2** Phenotype of different *atpds3* mosaic/chimeric mutants generated using different CRISPR/Cas9 vectors in T1 generation. **a** Phenotype of *atpds3* mosaic/chimeric mutant at the flowering stage with albino dominance with patchy green leaves. **b** Phenotype of *atpds3* mosaic/chimeric mutant at the flowering stage with complete albino branches and flowers. **c** Phenotype of *atpds3* mosaic/chimeric mutant with complete albino and green pods on the same stem. **d** Phenotype of *atpds3* mosaic/chimeric mutant with greenish dominant over albino leaves. Red arrows show the albino pod, flower buds or leaves.

**Figure S3**

**
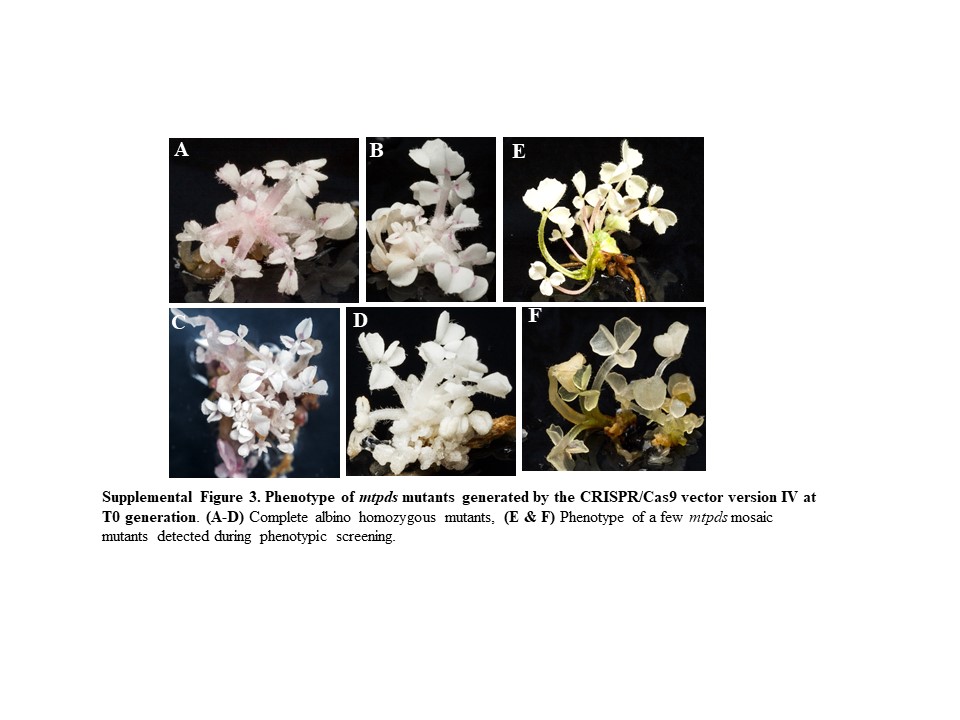
**

**Figure S3** Phenotype of *mtpds* mutants generated using the CRISPR/Cas9 vector version IV at T0 generation. **a-d** Complete albino homozygous mutants. **e and f** Phenotype of a few *mtpds* mosaic mutants detected during phenotypic screening.

**Figure S4**

**
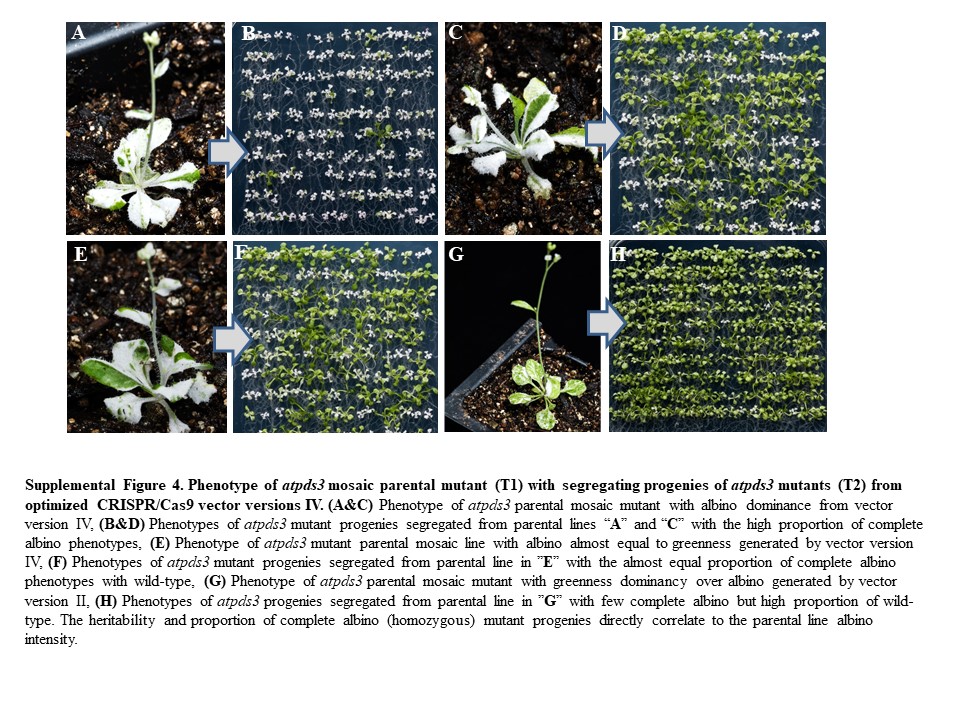
**

**Figure S4** Phenotype of *atpds3* mosaic parental mutant (T1) with segregating progenies of *atpds3* mutants (T2) from CRISPR/Cas9 vector version IV. **a and c** Phenotype of *atpds3* parental mosaic mutant with albino dominance from vector version IV. **b and** **d** Phenotypes of *atpds3* mutant progenies segregated from parental lines “a” and “c” with the high proportion of complete albino phenotypes. **e** Phenotype of *atpds3* mutant parental mosaic line with albino almost equal to greenness generated by vector version IV. **f** Phenotypes of *atpds3* mutant progenies segregated from parental line in ”e” with the almost equal proportion of complete albino phenotypes with wild-type. **g** Phenotype of *atpds3* parental mosaic mutant with greenness dominancy over albino generated by vector version II. **h** Phenotypes of *atpds3* progenies segregated from parental line in ”**g**” with few complete albino but high proportion of wild-type. The heritability and proportion of complete albino (homozygous) mutant progenies directly correlate to the parental line albino intensity.

**Figure S5**

**
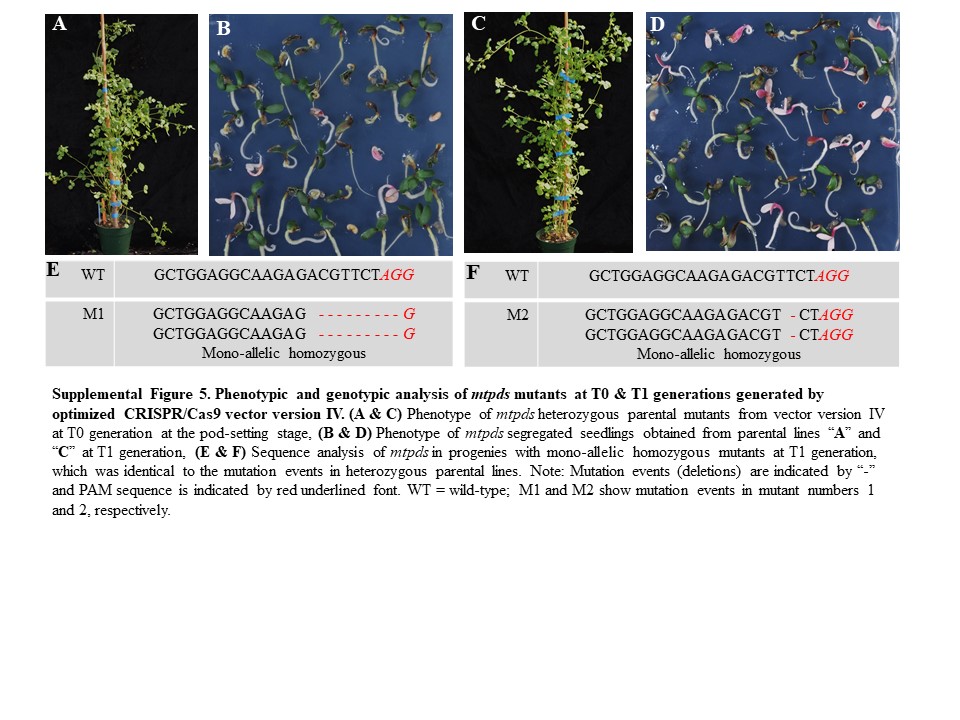
**

**Figure S5** Phenotypic and genotypic analysis of *mtpds* mutants at T0 & T1 generations generated using the CRISPR/Cas9 vector version IV. **a** and **c** Phenotype of *mtpds* heterozygous parental mutants from vector version IV at T0 generation at the pod-setting stage. **b** and **d** Phenotype of *mtpds* segregated seedlings obtained from parental lines “a” and “c” at T1 generation. **e** and **f** Sequence analysis of *mtpds* in progenies with mono-allelic homozygous mutants at T1 generation, which was identical to the mutation events in heterozygous parental lines. Note: Mutation events (deletions) are indicated by “-” and PAM sequence is indicated by red underlined font. WT = wild-type; M1 and M2 show mutation events in mutant numbers 1 and 2, respectively.

**Table S1 List of primers and gRNAs used in this study**

| Name | Sequence |
| --- | --- |
| AtPDS-gRNA-F | GGACTTTTGCCAGCCATGGTCGG |
| AtPDS-gRNA-R | CCGACCATGGCTGGCAAAAGTCC |
| MtPDS-gRNA-F | GCTGGAGGCAAGAGACGTTCT |
| MtPDS-gRNA-R | AGAACGTCTCTTGCCTCCAGC |
| 35S-F | ACAGTCTCAGAAGACCAAAGGGC |
| 35S-R | TGTTCTCTCCAAATGAAATGAAC |
| At UBQ10-F | CTGCAGGTCGACGAGTCAGTAATA |
| At UBQ10-R | TGTTAATCAGAAAAACTCAGATTAAT |
| EC1-2p-F | GAATAAAAGCATTTGCGTTTGG |
| EC1-2p-R | TAGATTTCTCAACAGATTGATAAG |
| AMGE3p-F | CGTGGCAATCGATACTTGCTA |
| AMGE3p-R | TTGCACACTCAAATCCTAAAA |
| AtU6-F | GTGATTGTGAGACCGAGAG |
| AtU6-R | CTGATAACTCTGATGTGGATAAG |
| Hyg-F | AAGGAATCGGTCAATACACTACATGG |
| Hyg-R | AAGACCAATGCGGAGCATATACG |
| PPT-F | GAAGTCCAGCTGCCAGAAAC |
| PPT-R | AGTCGACCGTGTACGTCTCC |
| AtPDS-RT-F | ACTCCGTTGTAGCATTAGC |
| AtPDS-RT-R | ATGCTAACTACTCTACCAAGT |
| MtPDS-RT-F | GTACCATATTATTGTGTTGGATAT |
| MtPDS-RT-R | GTTCAACATTACCACTCCAAT |
